# A predictive model in patients with chronic hydrocephalus following aneurysmal subarachnoid hemorrhage: a retrospective cohort study

**DOI:** 10.3389/fneur.2024.1366306

**Published:** 2024-05-16

**Authors:** Dai Rao, Li Yang, Xu Enxi, Lu Siyuan, Qian Yu, Li Zheng, Zhou Zhou, Chen Yerong, Chen Bo, Shan Xiuhong, Sun Eryi

**Affiliations:** ^1^Department of Radiology, Affiliated People’s Hospital of Jiangsu University, Zhenjiang, Jiangsu, China; ^2^Department of Neurosurgery, Affiliated People’s Hospital of Jiangsu University, Zhenjiang, Jiangsu, China

**Keywords:** clinical-radiological nomogram, chronic hydrocephalus, aneurysmal subarachnoid hemorrhage, white matter, CT scan

## Abstract

**Objective:**

Our aim was to develop a nomogram that integrates clinical and radiological data obtained from computed tomography (CT) scans, enabling the prediction of chronic hydrocephalus in patients with aneurysmal subarachnoid hemorrhage (aSAH).

**Method:**

A total of 318 patients diagnosed with subarachnoid hemorrhage (SAH) and admitted to the Department of Neurosurgery at the Affiliated People’s Hospital of Jiangsu University between January 2020 and December 2022 were enrolled in our study. We collected clinical characteristics from the hospital’s medical record system. To identify risk factors associated with chronic hydrocephalus, we conducted both univariate and LASSO regression models on these clinical characteristics and radiological features, accompanied with penalty parameter adjustments conducted through tenfold cross-validation. All features were then incorporated into multivariate logistic regression analyses. Based on these findings, we developed a clinical-radiological nomogram. To evaluate its discrimination performance, we conducted Receiver Operating Characteristic (ROC) curve analysis and calculated the Area Under the Curve (AUC). Additionally, we employed calibration curves, and utilized Brier scores as an indicator of concordance. Additionally, Decision Curve Analysis (DCA) was performed to determine the clinical utility of our models by estimating net benefits at various threshold probabilities for both training and testing groups.

**Results:**

The study included 181 patients, with a determined chronic hydrocephalus prevalence of 17.7%. Univariate logistic regression analysis identified 11 potential risk factors, while LASSO regression identified 7 significant risk factors associated with chronic hydrocephalus. Multivariate logistic regression analysis revealed three independent predictors for chronic hydrocephalus following aSAH: Periventricular white matter changes, External lumbar drainage, and Modified Fisher Grade. A nomogram incorporating these factors accurately predicted the risk of chronic hydrocephalus in both the training and testing cohorts. The AUC values were calculated as 0.810 and 0.811 for each cohort respectively, indicating good discriminative ability of the nomogram model. Calibration curves along with Hosmer-Lemeshow tests demonstrated excellent agreement between predicted probabilities and observed outcomes in both cohorts. Furthermore, Brier scores (0.127 for the training and 0.09 for testing groups) further validated the predictive performance of our nomogram model. The DCA confirmed that this nomogram provides superior net benefit across various risk thresholds when predicting chronic hydrocephalus. The decision curve demonstrated that when an individual’s threshold probability ranged from 5 to 62%, this model is more effective in predicting the occurrence of chronic hydrocephalus after aSAH.

**Conclusion:**

A clinical-radiological nomogram was developed to combine clinical characteristics and radiological features from CT scans, aiming to enhance the accuracy of predicting chronic hydrocephalus in patients with aSAH. This innovative nomogram shows promising potential in assisting clinicians to create personalized and optimal treatment plans by providing precise predictions of chronic hydrocephalus among aSAH patients.

## Introduction

Aneurysmal subarachnoid hemorrhage (aSAH) is a common cause of hemorrhagic stroke, affecting approximately 6 to 10.5 out of every 100,000 individuals (1). The mortality rate associated with aSAH is around 45%, and most survivors experience functional morbidity and neurocognitive deficits (2). Hydrocephalus often develops after aSAH and can result in extended hospital stays, increased medical costs, disability, and even death (3). It occurs in three stages: the acute phase (24–72 h after SAH) affects about 20% of cases; the subacute phase (between days 4 and 13) has an incidence of approximately 2–3%; and the chronic phase (after day 14) ranges from 7 to 48% (4). Acute or subacute secondary hydrocephalus can emerge rapidly as a severe complication during certain neurosurgical conditions. Although it may be masked by unconsciousness or subtle symptoms, timely clinical diagnosis and treatment are essential. However, chronic hydrocephalus is often underdiagnosed as one of the few reversible causes of dementia; it may occur in up to 14% of patients in nursing home settings (5). Due to masked symptoms by the patient’s condition, failure by medical staff to promptly identify and intervene could impact prognosis. Therefore, prompt intervention through timely identification of patients at risk for chronic hydrocephalus is crucial since placement of a shunt system improves clinical outcomes for individuals affected by aSAH (6).

Numerous investigations have been conducted to identify significant factors that can predict the development of chronic hydrocephalus following aSAH. These factors include intraventricular hemorrhage (IVH), aneurysm location (7), and modified Fisher scores on CT scans (8). Some studies suggest that risk elements linked to the patient’s initial clinical presentation include acute hydrocephalus, Hunt-Hess grade—a pivotal measure for evaluating neurological injury and consciousness level in aSAH patients (9), World Federation of Neurological Surgeons (WFNS) grade, age, and initial level of consciousness. Additionally, the technique employed for aneurysm repair, such as surgical intervention and intracranial infection (10), also exerts influence. However, these investigations have yielded varied outcomes.

Radiological imaging is crucial in diagnosing chronic hydrocephalus in adults, although its reliability as a predictive tool is still debated. Analyzing neuroimages for structural changes related to hydrocephalus can be accomplished through morphometric analysis. The study focused on traditional radiographic characteristics such as enlarged subarachnoid space, dilated temporal horns, and dilated third ventricle (11). In our research, various commonly used radiographic parameters were collected and compared, including frontal horn length, lateral length, Evans’ ratio, sellar bone distortion, empty sella [defined as at least 66% pituitary height loss (5)], cerebrospinal fluid (CSF) extravasation (12), third ventricle width (13), callosal angle, and tentorial angle (14, 15).

Given the debilitating impact of aSAH, it is often challenging to detect patients who develop chronic hydrocephalus and subsequently experience progressive nerve function deterioration following discharge (16). The limited universal applicability of lumbar puncture as an invasive diagnostic method for symptomatic patients leads to numerous undiagnosed cases, resulting in missed opportunities for optimal treatment (17).

Moreover, long-standing chronic hydrocephalus can lead to irreversible symptoms (18). Surgical interventions such as ventriculoperitoneal or ventriculoatrial shunt procedures have demonstrated significant efficacy in ameliorating chronic hydrocephalus-related symptoms (6, 19). Hence, there exists an urgent need to promptly identify high-risk patients for chronic hydrocephalus to enable early intervention. The development of predictive models that stratify aSAH patients based on their risk of developing chronic hydrocephalus is crucial. Data derived from these models could guide neurosurgeons toward earlier placement of shunt systems for higher-risk patients, ultimately leading to improved prognosis outcomes. Our current study endeavors to construct a nomogram utilizing both clinical and radiological characteristics to predict the occurrence of chronic hydrocephalus in aSAH patients. This tool holds substantial potential for enhancing the diagnosis and treatment strategies for individuals affected by this condition.

## Methods

### Study design and patients

**Figure 1:**
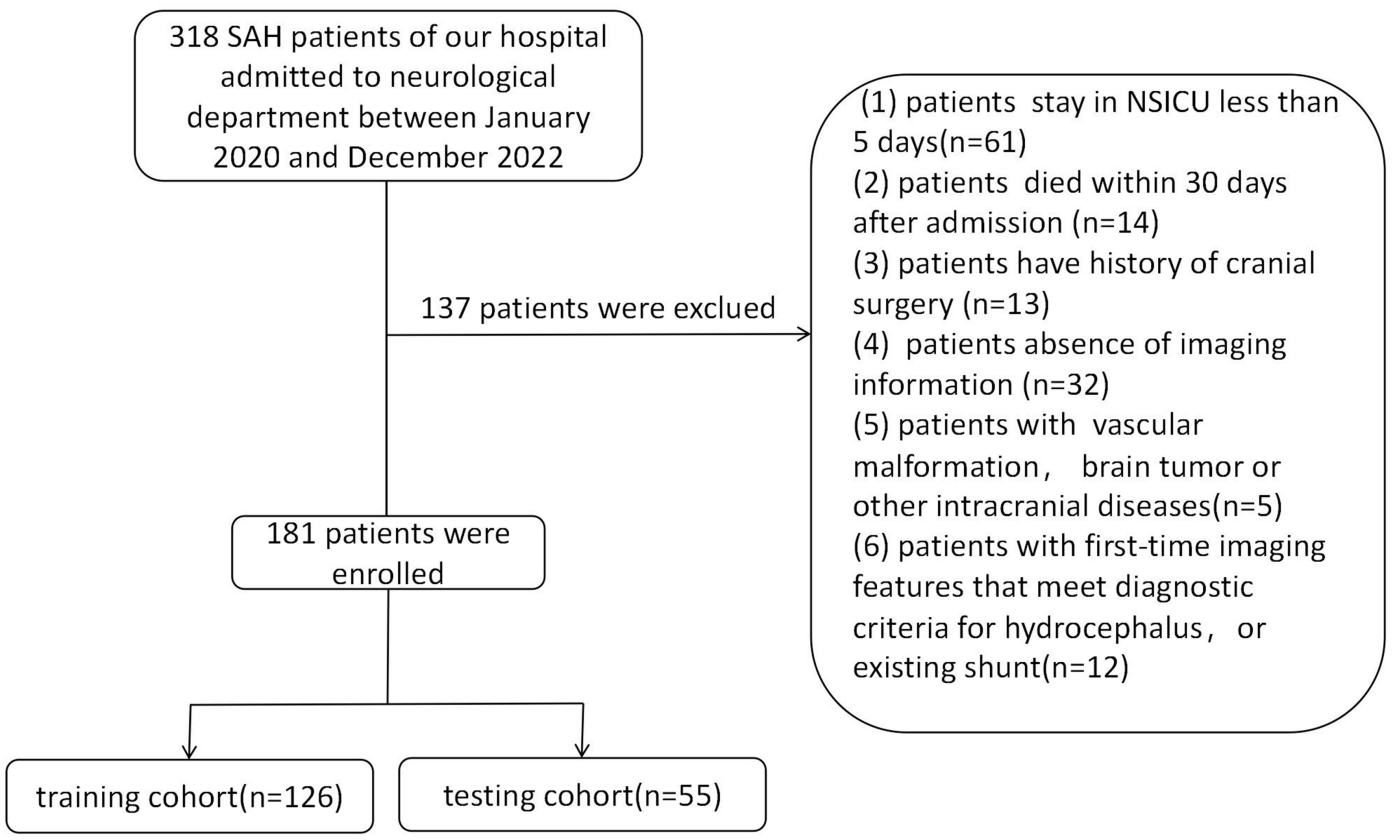
Recruitment pathway for eligible patients in this study.

Our study enrolled 318 patients with SAH who were admitted to the neurosurgery department at the Affiliated People’s Hospital of Jiangsu University between January 2020 and December 2022. Patient data were retrospectively collected from our clinical research data platform. Exclusion criteria included the absence of aneurysms, refusal or death within 30 days post-treatment, the presence of other cerebral diseases such as vascular malformation or brain tumor, and a history of hydrocephalus or existing shunt. After refining and extracting baseline data, statistical analysis was conducted on medical records from a total of 181 subjects. These patients were randomly divided into training and validation cohorts in a ratio of 7:3 (Figure 1). The retrospective study was approved by the Medical Ethics Committee of the Affiliated People’s Hospital of Jiangsu University (Approval No.: K-20220173-Y) and in accordance with the Declaration of Helsinki. The informed consent was not required, as all patient data underwent anonymization and de-identification before analysis.

### Data collection of patients

The patient demographics and clinical data, encompassing age, gender, orotracheal intubation, diagnosis, and surgical history, were meticulously extracted from the medical record system. Each piece of data underwent thorough validation by domain experts to ensure utmost precision and consistency. Serological tests were conducted within the initial week, with reference ranges as follows: white blood cell count: 3.5–9.5 × 10^9^/L; neutrophil percentage: 1.8–6.3 × 10^9^/L; C-reactive protein (CRP): 0–5 mg/L.

**Figure 2:**
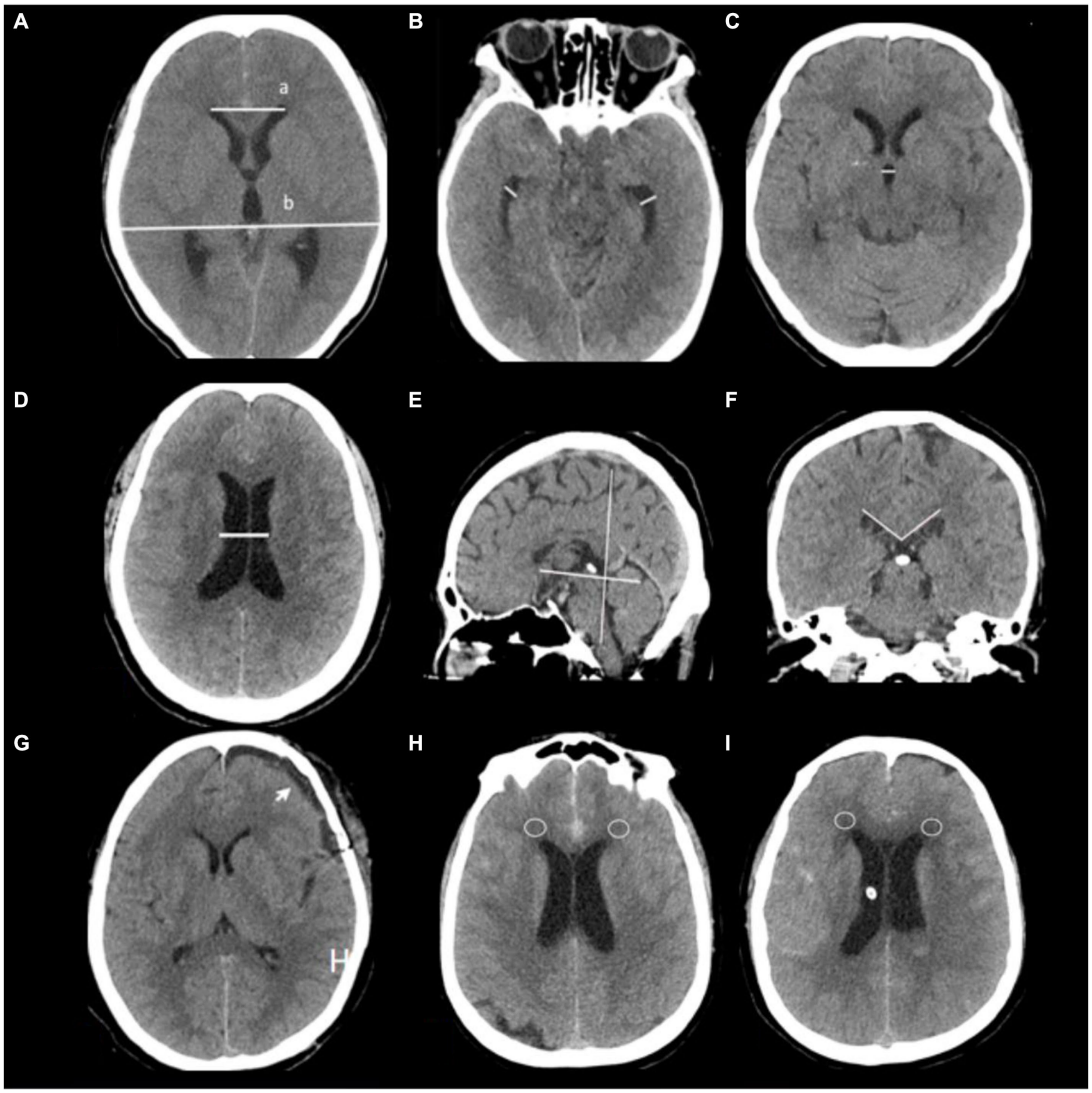
Radiological features are depicted in this study. **(A)** Evaluation of hydrocephalus entailed the use of CT scans and Evans’ index, which measures the ratio between the maximum width of frontal horns (a) and the maximal internal diameter of the skull (b) at Monro’s foramens. **(B)** Temporal horn widths are measured strictly at the ventricular prominence level. **(C)** Third ventricle width, **(D)** Ventricular width refers to the narrowest distance between walls on both sides of the lateral ventricle body, measured axially; CT slices should be taken where the cella media first appears unobscured by other regions (thalamus) **(G)** subdural hygroma after surgery (arrow). **(E,F)** Corpus callosal angles are measured on coronal planes perpendicular to each subject’s anteroposterior commissure plane on their posterior commissure. **(H,I)** CT values around anterior horns intervals a patient 7 days after admission (circles place).

Evaluation of hydrocephalus entailed the use of CT scans and Evans’ index, which measures the ratio between the maximum width of frontal horns and the maximal internal diameter of the skull at Monro’s foramens. A diagnosis of hydrocephalus is established when an Evans’ index value exceeds 0.30 (20). The distance between frontal horns is determined by measuring a continuous plane, while the maximum internal diameter in that same plane determines its former (21). Temporal horn widths are measured strictly at the ventricular prominence level, where their cavities should rarely exceed 1 mm in width; diameters greater than 2 mm in adults are considered pathological (12). Corpus callosal angles are measured on coronal planes perpendicular to each subject’s anteroposterior commissure plane on their posterior commissure; a callosal angle less than 90° supports suspected chronic hydrocephalus (22). Ventricular width refers to the narrowest distance between walls on both sides of the lateral ventricle body, measured axially; CT slices should be taken where the cella media first appears unobscured by other regions (thalamus) (23). Diffusion Tensor Imaging (DTI) has revealed significant increases in periventricular and deep cerebral white matter fraction anisotropy for hydrocephalus compared with healthy elderly individuals; CT scans may exhibit decreased density in deep white matter, known as leukoporosis (24). In this study, patients are identified as positive if they display reduced white matter density around anterior horns intervals 7 days after admission, wherein CT values decrease by ≥5 HU in diameter 8 mm. These radiological features are depicted in Figure 2.

The comprehensive compilation included detailed medical records, complications, distinctive clinical characteristics, as well as academic as well as clinical indicators. Patient consciousness was assessed using the Glasgow Coma Scale (GCS) score, while patient condition was evaluated by selecting the lowest score from the Hunt Hess and World Federation of Neurological Surgeons Scale for categorization.

### Selection of risk factors and construction of the nomogram

The study employed univariate logistic regression methods to analyze clinical parameters, while incorporating all attributes into the LASSO regression analysis with penalty parameter adjustments conducted through tenfold cross-validation. Following this, multivariate logistic regression techniques were used to identify significant risk factors for chronic hydrocephalus after aSAH among positive characteristics identified from both LASSO and univariate logistic analyses, as previously explained (25). These factors were then combined using multivariate logistic regression in the primary cohort, resulting in the creation of a user-friendly nomogram for healthcare professionals.

### Statistical analysis

Statistical analyses were conducted using SPSS 22.0 and R software (version 4.1) (25). Continuous variables were expressed as median (interquartile range) or mean ± standard deviation (SD), depending on the outcome of the Shapiro–Wilk test. Conversely, categorical data were presented as proportions. Disparities across cohorts were discerned through the application of the Chi-squared test, Fisher’s exact test, and Mann–Whitney U test. Logistic regression was utilized to pinpoint risk factors associated with study outcomes, considering variables with a *p*-value <0.05 in univariate logistic regression analysis for both step-down logistic regression models. The nomogram formulation involved the implementation of the regression model exhibiting the minimum Akaike’s information criterion (AIC). The performance of the nomogram was appraised by calculating the area under the receiver operating characteristic (ROC) curve (AUC) and comparing predicted versus observed incidences of outcomes. Calibration curves assessed fidelity followed by a goodness-of-fit examination. The calibration curve performance was evaluated using Brier score. Furthermore, the clinical utility of refined models at various probability thresholds for both cohorts was evaluated through decision curve analysis (DCA). A *p*-value <0.05 indicated statistical significance.

## Results

### Patient characteristics

A retrospective analysis was undertaken utilizing data from 318 patients enrolled at our institution, with 181 patients included in this study. This cohort was randomly divided into two groups, earmarked for training and testing, at a ratio of 7:3 (Figure 1). There were no significant differences observed in all variables between the training and testing groups (Table 1, *p* > 0.05). The incidence rate of chronic hydrocephalus was calculated at 17.7% (32/181), manifesting as 16.7% (21/126) within the training group and 20.0% (11/55) within the testing group.

**Table 1:** The baseline characteristics of the enrolled patients in the training and validation cohorts.

	[ALL] *N*=181	Test *N*=55	Train *N*=126	*p*. overall
Group	0.18 (0.38)	0.11 (0.31)	0.21 (0.41)	0.083
Gender, *n* (%)				0.622
Female	122 (67.4%)	39 (70.9%)	83 (65.9%)	
Male	59 (32.6%)	16 (29.1%)	43 (34.1%)	
Age (years) Media[Q1;Q3]	59.0 [52.0;67.0]	58.0 [52.0;66.5]	60.0 [53.0;68.0]	0.488
Past medical history				
Ischemic stroke, *n* (%)				0.316
Negative	177 (97.8%)	55 (100%)	122 (96.8%)	
Positive	4 (2.21%)	0 (0.00%)	4 (3.17%)	
Hypertension, *n* (%)				0.170
Negative	93 (51.4%)	33 (60.0%)	60 (47.6%)	
Positive	88 (48.6%)	22 (40.0%)	66 (52.4%)	
Heart Disease, *n* (%)				0.202
Negative	174 (96.1%)	51 (92.7%)	123 (97.6%)	
Positive	7 (3.87%)	4 (7.27%)	3 (2.38%)	
Diabetes, *n* (%)				0.757
Negative	169 (93.4%)	51 (92.7%)	118 (93.7%)	
Positive	12 (6.63%)	4 (7.27%)	8 (6.35%)	
Surgical method, *n* (%)				0.377
Coiling	98 (54.1%)	33 (60.0%)	65 (51.6%)	
Clipping	83 (45.9%)	22 (40.0%)	61 (48.4%)	
Craniectomy, *n* (%)				0.223
Negative	158 (87.3%)	45 (81.8%)	113 (89.7%)	
Positive	23 (12.7%)	10 (18.2%)	13 (10.3%)	
Location of aneurysm, *n* (%)				0.687
Anteria	22 (12.2%)	8 (14.5%)	14 (11.1%)	
Posterior	159 (87.8%)	47 (85.5%)	112 (88.9%)	
External lumbar drainage, *n* (%)				1.000
Negative	154 (85.1%)	47 (85.5%)	107 (84.9%)	
Positive	27 (14.9%)	(14.5%)	19 (15.1%)	
Hunt and Hess Grade, Media[Q1;Q3]	2.00 [1.00;3.00]	2.00 [1.00;2.50]	2.00 [1.00;3.00]	0.294
GCS score, Media[Q1;Q3]	15.0 [12.0;15.0]	15.0 [13.0;15.0]	15.0 [12.0;15.0]	0.458
WFNS Grade, Media[Q1;Q3]	1.00 [1.00;4.00]	1.00 [1.00;2.00]	1.00 [1.00;4.00]	0.468
Acute hydrocephalus, *n* (%)				0.508
Negative	170 (93.9%)	53 (96.4%)	117 (92.9%)	
Positive	11 (6.08%)	2 (3.64%)	9 (7.14%)	
Subacute hydrocephalus, *n* (%)				1.000
Negative	172 (95.0%)	52 (94.5%)	120 (95.2%)	
Positive	9 (4.97%)	3 (5.45%)	6 (4.76%)	
EVD placement, *n* (%)				
Negative	157 (86.7%)	47 (85.5%)	110 (87.3%)	
Positive	24 (13.3%)	8 (14.5%)	16 (12.7%)	
V-P shunt, *n* (%)				1.000
Negative	177 (97.8%)	54 (98.2%)	123 (97.6%)	
Positive	4 (2.21%)	1 (1.82%)	3 (2.38%)	
Callosal Angle, Media[Q1;Q3]	111 (9.09)	111 (9.19)	111 (9.09)	0.783
Intracranial infection, *n* (%)				0.304
Negative	180 (99.4%)	54 (98.2%)	126 (100%)	
Positive	1 (0.55%)	1 (1.82%)	0 (0.00%)	
Intraventricular bleeding, *n* (%)				0.113
Negative	111 (61.3%)	39 (70.9%)	72 (57.1%)	
Positive	70 (38.7%)	16 (29.1%)	54 (42.9%)	
Modified Fisher Grade, *n* (%)				0.622
1	54 (29.8%)	16 (29.1%)	38 (30.2%)	
2	74 (40.9%)	26 (47.3%)	48 (38.1%)	
3	46 (25.4%)	11 (20.0%)	35 (27.8%)	
4	7 (3.87%)	2 (3.64%)	5 (3.97%)	
Interval to blood clearance (days), Media[Q1;Q3]	15.0 [11.0;25.0]	15.0 [10.5;25.0]	16.0 [12.0;24.8]	0.433
The distance between frontal horns (mm), Media[Q1;Q3]	33.0 [31.0;36.3]	32.5 [30.9;37.9]	33.2 [31.0;36.0]	0.984
Evans index, Media[Q1;Q3]	0.26 [0.24;0.28]	0.26 [0.24;0.29]	0.26 [0.24;0.28]	0.735
Left Temporal Horn (mm), Media[Q1;Q3]	3.61 [3.00;5.00]	4.00 [3.00;5.00]	3.00 [2.45;5.00]	0.130
Right Temporal Horn (mm), Media[Q1;Q3]	3.13 [2.86;5.00]	4.00 [3.00;5.17]	3.00 [2.42;4.92]	0.119
Third ventricle width (mm), Media[Q1;Q3]	6.00 [5.00;8.00]	6.00 [5.00;7.80]	6.00 [4.86;8.00]	0.451
The narrowest width between the lateral walls (mm), Media[Q1;Q3]	26.0 [22.0;31.0]	25.0 [22.0;30.0]	27.0 [22.0;31.0]	0.538
Sellar bone distortion, *n* (%)				0.071
Negative	136 (75.1%)	36 (65.5%)	100 (79.4%)	
Positive	45 (24.9%)	19 (34.5%)	26 (20.6%)	
Periventricular white matter changes, *n* (%)				0.076
Negative	123 (68.0%)	43 (78.2%)	80 (63.5%)	
Positive	58 (32.0%)	12 (21.8%)	46 (36.5%)	
Subdural hygroma, *n* (%)				0.200
Negative	128 (70.7%)	43 (78.2%)	85 (67.5%)	
Positive	53 (29.3%)	12 (21.8%)	41 (32.5%)	
CRP (mg/L), Media[Q1;Q3]	2.63 [1.13;5.89]	2.21 [0.90;6.91]	2.71 [1.24;5.73]	0.724
WBC count (10^9^/L), Media[Q1;Q3]	10.1 [8.10;13.0]	10.3 [8.85;13.1]	9.85 [8.00;13.0]	0.428
Neutrophil (10^9^/L), Media[Q1;Q3]	8.50 [6.40;11.6]	9.10 [6.85;11.7]	8.40 [6.20;11.3]	0.504

### Selection of risk factors and construction of the nomogram

**Figure 3:**
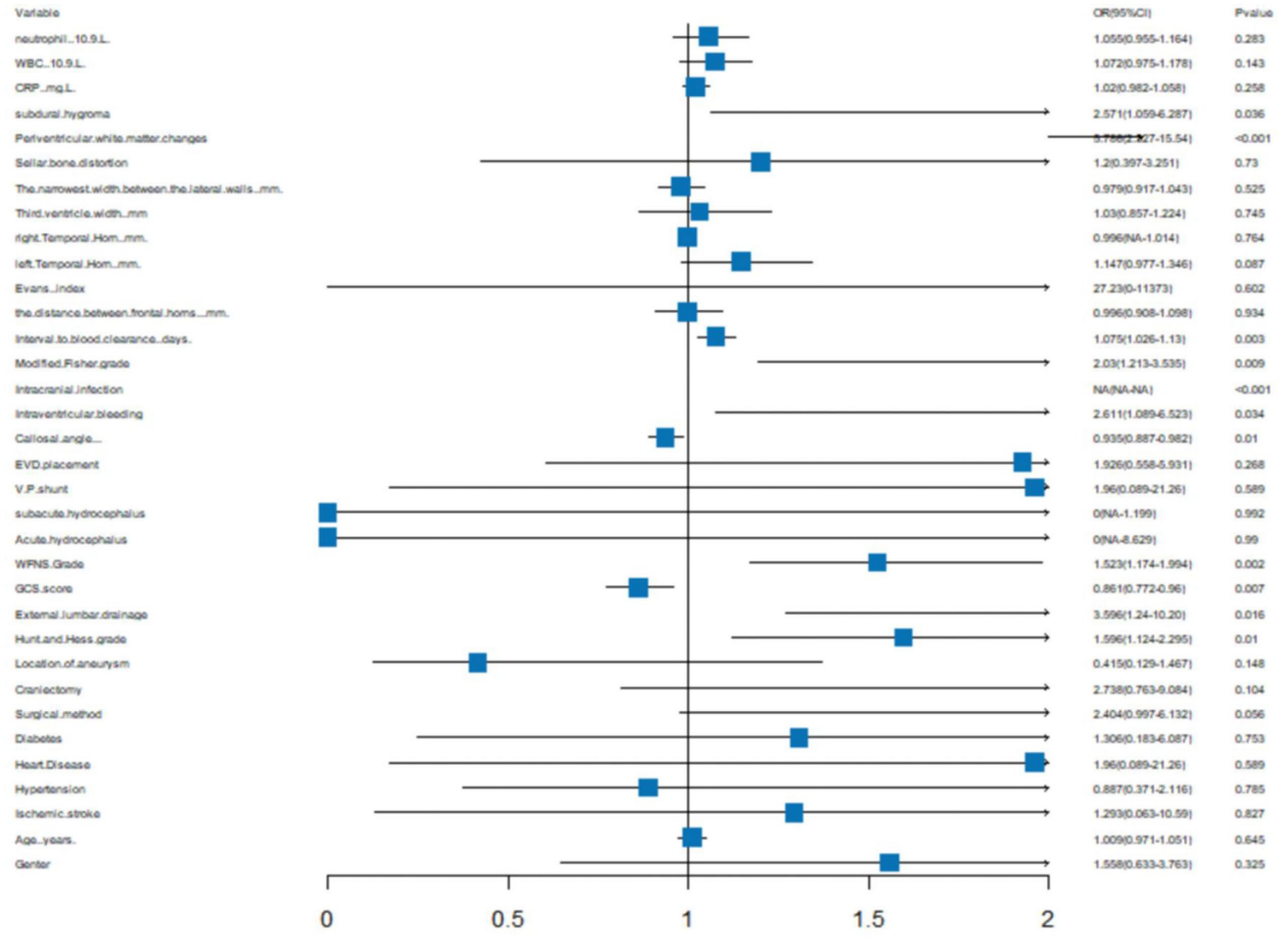
A forest image to show all of the characteristics among Univariatelogistic regression analyses.

**Figure 4:**
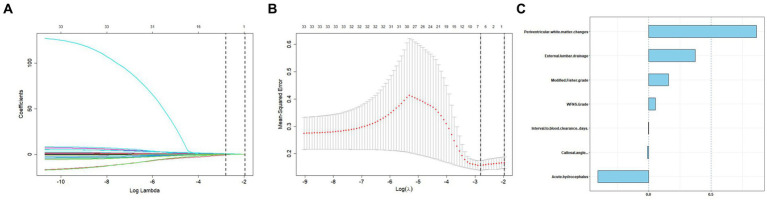
Tuning parameter selection using the LASSO regression in the training cohort. **(A)** LASSO coefficient profiles of the clinical features. **(B)** The optimal penalization coefficient lambda was generated in the LASSO via 10-fold cross-validation. The lambda value of the 1-fold mean square error for the training cohort was given. **(C)** All of the positive characteristics among LASSO.

**Figure 5:**
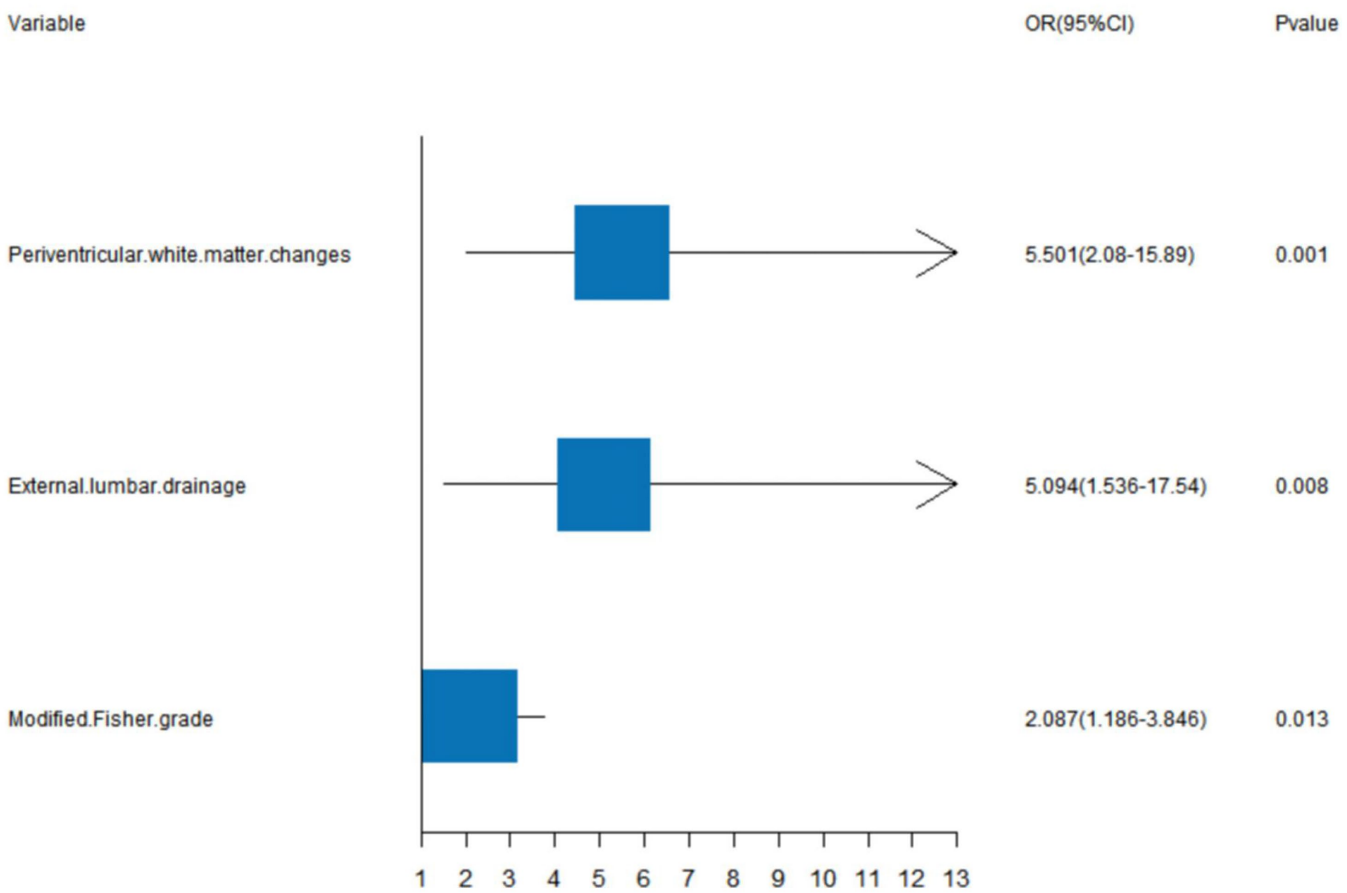
A forest image to show positive characteristics among multivariate logistic regression analyses.

**Figure 6:**
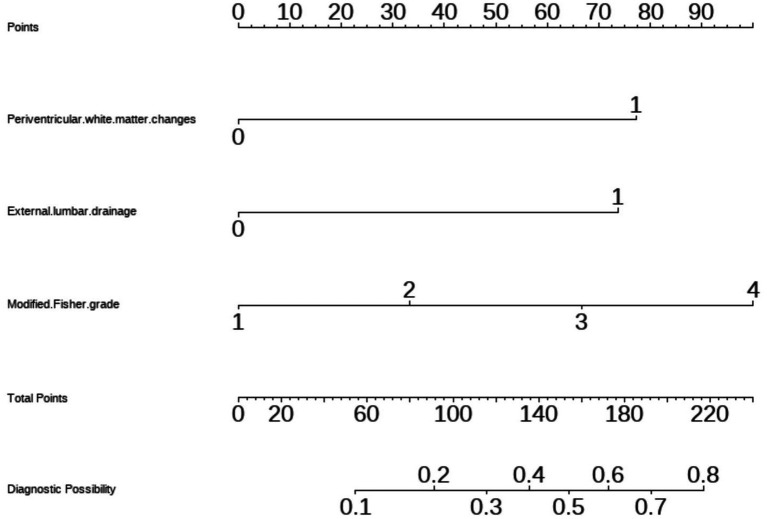
Nomogram based on multivariate logistic regression analysis for the prediction of happen of chronic hydrocephalus in aSAH patients.

We generated a forest plot to visually illustrate various risk factors associated with chronic hydrocephalus following aSAH using univariate logistic regression analyses (Figure 3). Subsequently, we identified 11 risk factors exhibiting a *p*-value <0.05 and implemented LASSO regression to identify seven prominent risk factors linked to chronic hydrocephalus after aSAH (Figure 4). Upon conducting multivariate logistic regression analyses (Table 2), we isolated three determinants for chronic hydrocephalus after aSAH: periventricular white matter changes, external lumbar drainage, and modified Fisher Grade. To depict these relationships, a forest plot was constructed (Figure 5). Furthermore, we developed a nomogram integrating the three identified factors to predict the probability of developing chronic hydrocephalus after aSAH, as depicted in Figure 6. To estimate an individual’s likelihood of experiencing chronic hydrocephalus after aSAH, each determinant in the nomogram was assigned a proportional score ranging from 0 to 100 based on its respective regression coefficient relative to chronic hydrocephalus after aSAH. The cumulative score was derived by summing up the scores of each determinant through drawing perpendicular lines from each determinant axis to the points axis on the nomogram. Finally, aligning this aggregate score on the total score scale provided an estimation of chronic hydrocephalus probability.

**Table 2:** Results of univariate and multivariate logistic regression analyses.

Characteristics	Univariate analysis				Multivariate analysis			
	*β* coefficient	OR	95% CI	*p*-value	*β* coefficient	OR	95% CI	*p*-value
Gender	0.444	1.558	1.558(0.633–3.763)	0.325				
Age	0.009	1.009	1.009(0.971–1.051)	0.645				
Ischemic stroke	0.257	1.293	1.293(0.063–10.59)	0.827				
Hypertension	−0.12	0.887	0.887(0.371–2.116)	0.785				
Heart disease	0.673	1.96	1.96(0.089–21.26)	0.589				
Diabetes	0.267	1.306	1.306(0.183–6.087)	0.753				
Surgical method	0.877	2.404	2.404(0.997–6.132)	0.056				
Craniectomy	1.007	2.738	2.738(0.763–9.084)	0.104				
Location of aneurysm	−0.879	0.415	0.415(0.129–1.467)	0.148				
Hunt and Hess Grade	0.467	1.596	1.596(1.124–2.295)	0.01				
External lumbar drainage	1.28	3.596	3.596(1.24–10.20)	0.016	1.628	5.094	5.094 (1.535–17.54)	0.008
GCS score	−0.149	0.861	0.861(0.772–0.96)	0.007				
WFNS Grade	0.421	1.523	1.523(1.174–1.994)	0.002				
Acute hydrocephalus	−16.313	0	0(NA-8.629)	0.99				
subacute hydrocephalus	−16.281	0	0(NA-1.199)	0.992				
V-P shunt	0.673	1.96	1.96(0.089–21.26)	0.589				
EVD placement	0.656	1.926	1.926(0.558–5.931)	0.268				
Callosal angle	−0.067	0.935	0.935(0.887–0.982)	0.01				
Intraventricular bleeding	0.96	2.611	2.611(1.089–6.523)	0.034				
Intracranial infection	−1.347		NA(NA-NA)	0				
Modified Fisher grade	0.708	2.03	2.03(1.213–3.535)	0.009	0.736	2.087	2.087 (1.185–3.846)	0.013
Interval to blood clearance	0.072	1.075	1.075(1.026–1.13)	0.003				
The distance between frontal horns	−0.004	0.996	0.996(0.908–1.098)	0.934				
Evans index	3.304	27.234	27.23(0–11,373)	0.602				
Left temporal horn	0.137	1.147	1.147(0.977–1.346)	0.087				
Right temporal horn	−0.004	0.996	0.996(NA-1.014)	0.764				
Third ventricle width	0.029	1.03	1.03(0.857–1.224)	0.745				
The narrowest width between the lateral walls	−0.021	0.979	0.979(0.917–1.043)	0.525				
Sellar bone distortion	0.182	1.2	1.2(0.397–3.251)	0.73				
Periventricular white matter changes	1.755	5.786	5.786(2.327–15.54)	<0.01	1.705	5.501	5.501 (2.079–15.89)	0.001
Subdural hygroma	0.944	2.571	2.571(1.059–6.287)	0.036				
CRP	0.02	1.02	1.02(0.982–1.058)	0.258				
WBC	0.07	1.072	1.072(0.975–1.178)	0.143				
Neutrophil	0.054	1.055	1.055(0.955–1.164)	0.283				

### Model evaluation and clinical application

**Figure 7:**
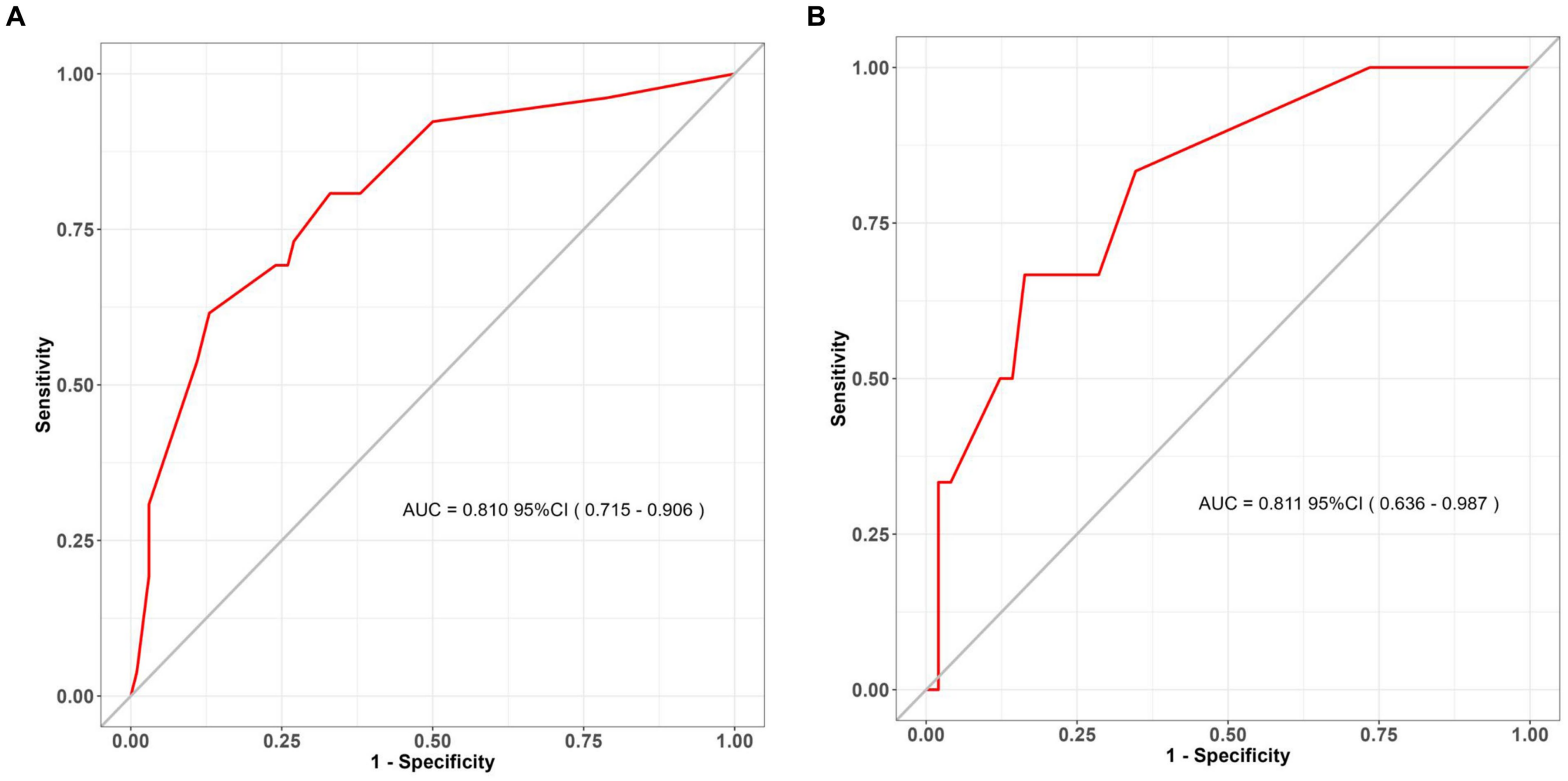
ROC curves for the assessment of discrimination performance of the clinical nomogram. ROC receiver operating characteristic. **(A)** The training set; **(B)** the validation set (bootstrap replicates = 500 times).

**Figure 8:**
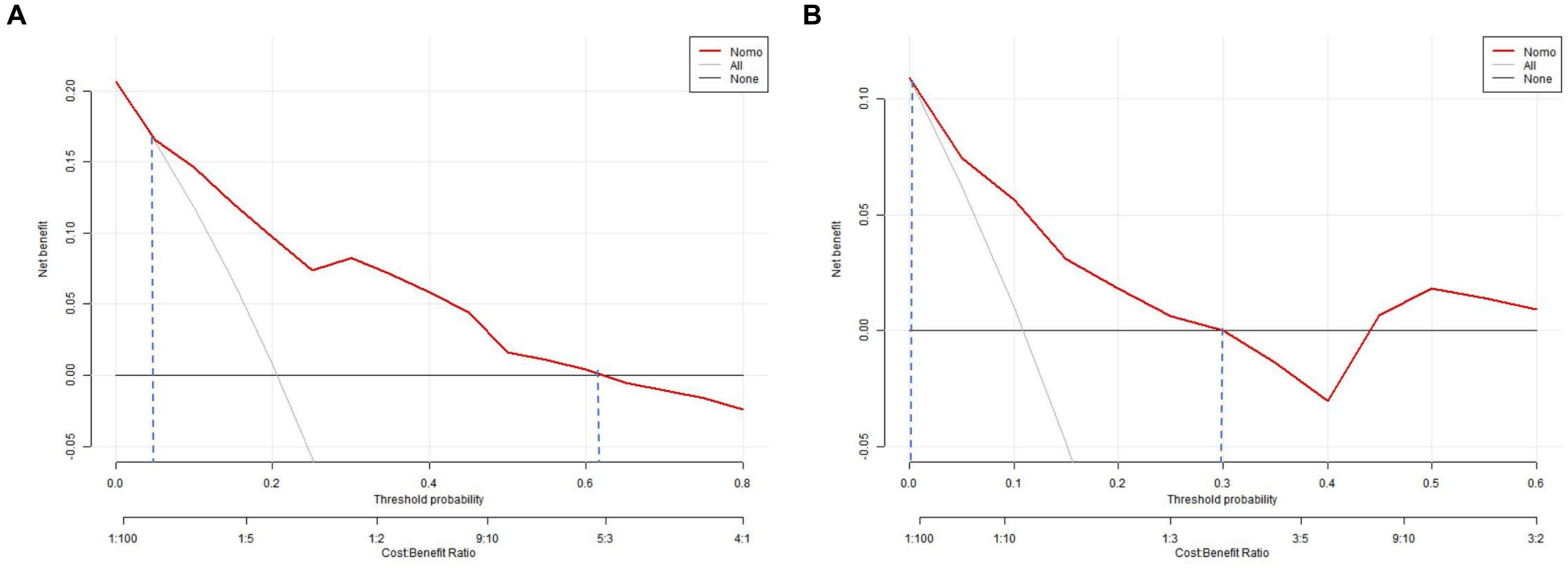
Calibration curve analysis for the nomogram in the panel **(A)** the training set and **(B)** the validation set.

**Figure 9:**
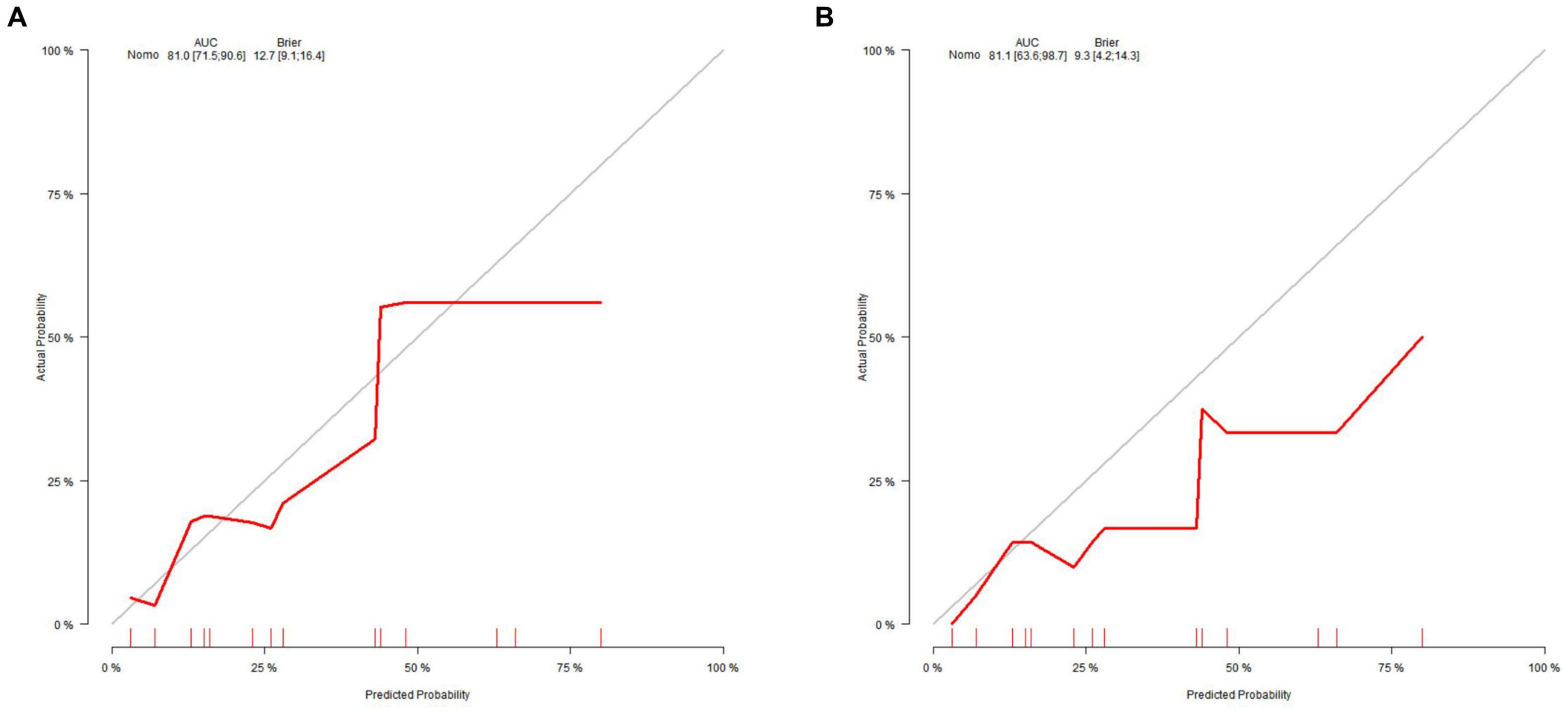
Decision curve analysis of the nomogram. **(A)** The training set; **(B)** the validation set. The decision curve shows that if the threshold probability of an individual is 5–62%, using this model to predict happen of choronic hydrocephalus after aSAH is better.

**Figure 10:**
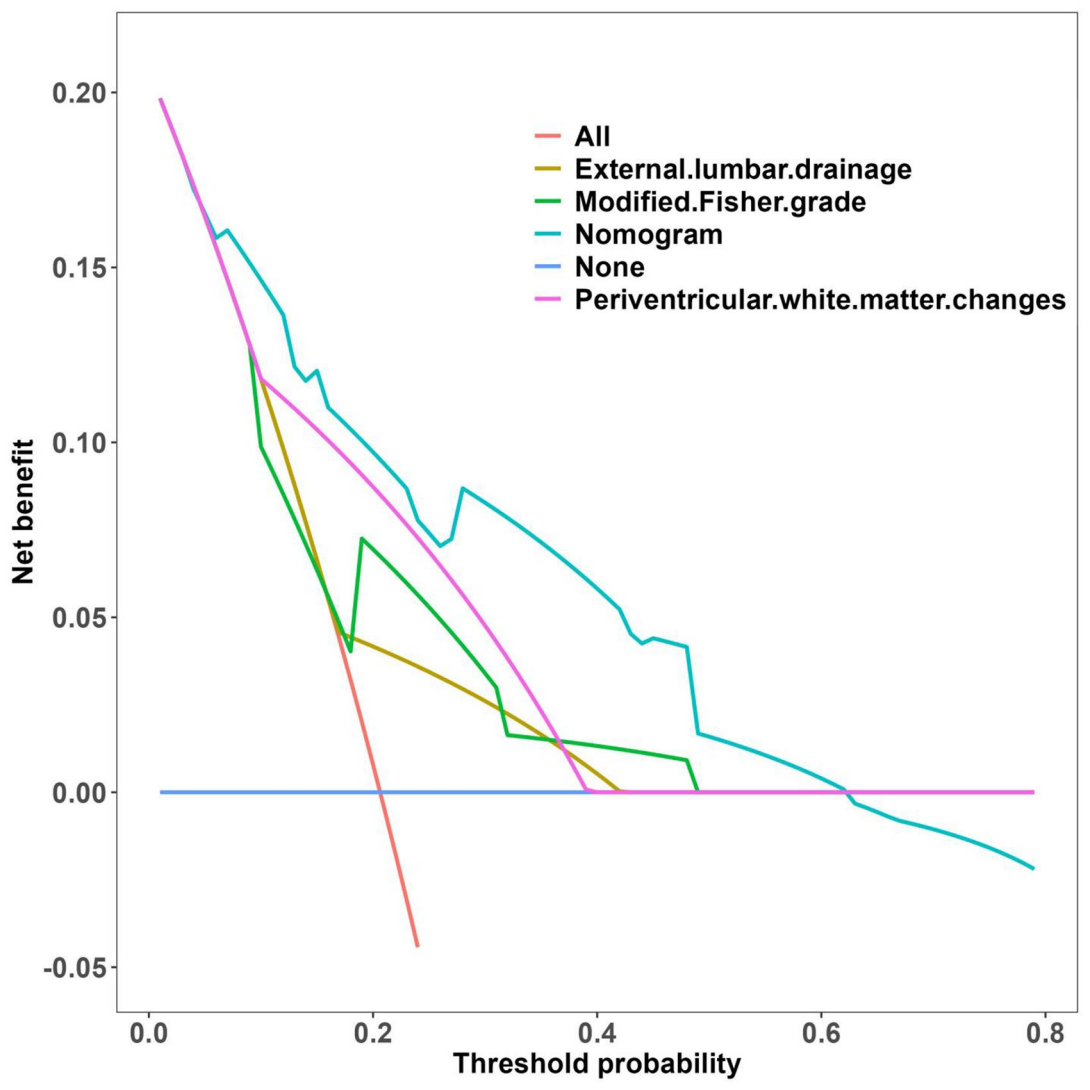
Decision curve analysis of all the positive characteristics.

The clinical-radiological nomogram underwent ROC analyses to predict the incidence of chronic hydrocephalus subsequent to aSAH in both study cohorts. Notably, the AUC values for the training and testing groups were 0.810 (95% CI: 0.715–0.906) and 0.811 (95% CI: 0.636–0.987), respectively, as delineated in Figure 7. Furthermore, Brier scores were 0.127 (95% CI: 0.091–0.164) for the training group and 0.09 (95% CI: 0.042–0.143) for the testing group, indicating strong agreement between the predictions of the nomogram and actual requirements for chronic hydrocephalus post-aSAH, as evidenced by analysis of the calibration curve presented in Figure 8. DCA highlighted the advantages of this model in predicting the occurrence of chronic hydrocephalus after aSAH, particularly when individual threshold probabilities ranged from 5 to 62%, as illustrated in Figure 9. Moreover, the DCA demonstrated that the nomogram offered superior net advantage over other risk thresholds in predicting needs for chronic hydrocephalus after aSAH, surpassing all alternatives, as depicted in Figure 10.

## Discussion

This study has yielded a clinical and radiological nomogram, offering an invaluable tool for precisely predicting chronic hydrocephalus in individuals diagnosed with aSAH. This nomogram laid a foundation for frontline healthcare practitioners to evaluate chronic hydrocephalus and develop personalized treatment plans. Crucially, the model’s ROC analysis demonstrated exceptional discriminatory and calibration capabilities, evident in an AUC value of 0.810 (95% CI: 0.715–0.906). Furthermore, DCA revealed substantial net clinical benefit associated with employing this model in both training and validation cohorts.

Various mechanisms have been proposed to contribute to the development of chronic hydrocephalus following aSAH, including impediments to CSF flow due to blood clot and scar tissue formation, compromised CSF absorption at the arachnoid granulations, and heightened CSF production by the choroid plexus arising from inflammation (26).

Notably, patients with a high Modified Fisher grade, indicative of increased blood in arachnoid debris, face a heightened risk of developing chronic hydrocephalus (27). The presence of arachnoid debris impedes CSF reabsorption caused by breakdown of blood components from leptomeningeal reactions (28), thereby fostering chronic hydrocephalus. These breakdown processes trigger an upsurge in pro-inflammatory cytokines, fibroblasts, increased collagen production, and toxic effects from iron accumulation during the post-aneurysm rupture weeks (29). Corroborating our findings, a high Modified Fisher grade emerges as a pivotal radiological factor predicting chronic hydrocephalus occurrence (29).

Furthermore, the potential clearance of blood in basal cisterns has been posited to mitigate chronic hydrocephalus post-Asah (27, 30). The aSAH disrupts CSF circulation at various sites, including the basal cisterns, ventricles, foramen of Monro, and extensive subarachnoid space (31). Accumulated blood and ensuing inflammatory processes can constrict the aqueduct’s diameter, impeding CSF circulation and thus fostering chronic hydrocephalus (32). Past studies have indicated that a substantial amount of subarachnoid and ventricular blood correlates with acute hydrocephalus necessitating external ventricular drainage (EVD) treatment (33). Our preliminary findings support the notion that EVD might not efficiently drain blood from central and peripheral CSF spaces (34), potentially delaying blood clearance. This delay could contribute to the development of chronic shunt-dependent hydrocephalus, as stagnant blood in these areas persists due to gravity while lighter CSF is drained (34). Indeed, EVD placement is imperative for managing acute hydrocephalus following aneurysm rupture; subsequent ventriculoperitoneal (VP) shunt insertion is required for up to 48% of patients (35). Lumbar drainage has been used to facilitate the rapid clearance of blood from the basal cisterns following aSAH. Two randomized clinical trials (36, 37) have shown that draining CSF via lumbar drainage following aSAH could decrease the incidence of delayed ischemic neurological deficits and improve early clinical outcomes through the rapid clearance of blood. However, there have been no high-quality trails exploring the relationship between lumbar drainage and chronic hydrocephalus. Our study, in fact, indicated that lumbar drainage increased the likelihood of chronic hydrocephalus. Chronic hydrocephalus has been demonstrated to be associated with the arachnoid detritus, formed by the breakdown of blood components due to leptomeningeal reactions and the volume of drained CSF (4). Although lumbar drainage could aid in blood clearance, potentially reducing chronic hydrocephalus (38), numerous studies have identified that CSF volumes exceeding 1,500 mL within the initial week of drainage (8), or high thresholds ranging from 78 to 214 mL of CSF drainage within 72 h after aSAH, serve as reliable predictors of chronic hydrocephalus (30). Consistent with our findings, lumbar drainage likely accelerates the removal of disintegrated blood products. However, in our department, CSF outflow consistently surpasses 214 mL within 72 h (30), with lumbar drainage durations exceeding 7 days, potentially elevating the incidence of shunt-dependent hydrocephalus. Thus, further research on the timing of lumbar drainage placement and the volume of CSF is warranted.

Some scholars have posited that microsurgical clipping may result in a heightened incidence of chronic hydrocephalus, hypothesizing that intraoperative manipulation of small vessels disrupts CSF homeostasis (33). Furthermore, certain studies have suggested that craniotomy could compromise the integrity of the cranial cavity, potentially leading to more profound disturbances in CSF circulation and an increased likelihood of chronic hydrocephalus (39). Additionally, subdural hygroma has been identified as a phenomenon influencing changes in CSF dynamics and may manifest prior to the onset of chronic hydrocephalus (40). Consequently, the avoidance of brain retraction may be imperative in mitigating the risk of chronic hydrocephalus (41). However, diverging conclusions have been drawn, as some have inferred that the hazard of chronic hydrocephalus is more pronounced with endovascular coiling than with clipping procedures. Our findings concur with reported data suggesting that the treatment modality does not serve as a prognostic variable for the development of chronic hydrocephalus (42).

At present, there exists an ongoing discourse concerning the impact of aneurysm treatment modalities (microsurgical clipping versus endovascular coiling) on the susceptibility to chronic hydrocephalus. Notably, some researchers have proposed that microsurgical clipping might lead to an elevated incidence of chronic hydrocephalus due to potential disturbances in CSF balance during the manipulation of small vessels in surgery (33). Studies suggest that craniotomy may compromise the cranial cavity’s integrity, leading to more pronounced disruptions in CSF circulation and an elevated likelihood of chronic hydrocephalus (39). The emergence of subdural hygroma has been identified as a phenomenon influencing shifts in CSF dynamics and may precede chronic hydrocephalus onset. To minimize the risk of chronic hydrocephalus development (40), avoiding brain retraction during surgical procedures may be essential (41). Conversely, contrasting evidence suggests that endovascular coiling poses a greater risk for chronic hydrocephalus compared to clipping procedures (26). Our research aligns with existing evidence indicating that treatment modality selection does not serve as a predictive factor for chronic hydrocephalus development (42).

The primary cause of neurological deficits in hydrocephalus arises from increased pressure on periventricular neural structures due to excessive CSF accumulation (24). Although relative ventricular size measurements have served as an objective radiological evaluation tool, they do not accurately gage hydrocephalus impact on periventricular neural structures (24). Radiological imaging reveals changes in the periventricular and deep white matter, which may be attributed to chronic ischemia or edema associated with hydrocephalus (43). Previous studies interpret these findings as stemming from mechanical pressure exerted by enlarged ventricles, causing stretching and compression of brain tissue. Increasing pressure triggers gradual brain tissue degeneration, resulting in stretched neural fibers, increased water diffusivity along axons, and decreased integrity of crossing fibers. The periventricular white matter displays progressive axonal degradation and demyelination (44, 45), influencing vital structures involved in motor control, sensory processing, and memory function such as the corpus callosum, internal capsule, fornix, and periventricular projection axons (46). Hence, diffusion tensor imaging (DTI) has been recommended as a valuable diagnostic tool for stroke patients with hydrocephalus (47), particularly in assessing fractional anisotropy (FA) values within the periventricular white matter adjacent to the anterior horn as a potential diagnostic biomarker (24, 48). It’s important to note that compared to other neural structures within the periventricular region, hydrocephalus appears to significantly compress the anterior corona radiata (49). Additionally, despite dissolution of blood clots within the ventricles, blood components infiltrate brain tissue and may negatively impact its functional structures. Smaller clots might obstruct the perivascular space, impeding the flow of CSF and interstitial fluid through the glymphatic system. These clots, alongside blood cells and their derivatives, disrupt the blood–brain barrier from within and weaken neurovascular unit functionality, fostering serum protein accumulation in ISF, particularly in the perivascular space, exacerbating their impact on the neuronal microenvironment. Ultimately, these changes affect arterial granulations (Pacchionian), leading to obstruction and inadequate functioning culminating in malresorptive hydrocephalus (50). In relation to our research, the periventricular white matter changes observed within a week indicate varying levels of interstitial water content, as evidenced by corresponding signal intensities in the periventricular white matter (19). Our findings were consistent with recent studies demonstrating reduced clearance of interstitial fluid in the periventricular region, accompanied by CSF reflux and congestion toward the deep white matter in individuals with hydrocephalus (51, 52).

While it may be intriguing to include variables related to infection (53), our study faced limitations due to a restricted sample size, necessitating larger sample sizes to prevent overfitting models and ensure consistent results when examining the impact of these prognostic factors. The retrospective nature of our study and its single-center design with a small sample size and only a 1-month follow-up period further constrained generalizability. It’s crucial to consider potential variations in treatment modalities, EVD weaning criteria, and decisions regarding VP shunt insertion among neurosurgeons. Moreover, the exclusion of patients developing delayed hydrocephalus requiring VPS implantation after 3 months, as reported in some studies (54). Presented an additional limitation. Lastly, the absence of external validation of our model left uncertainty regarding the generalizability of our findings.

## Conclusion

Our innovative nomogram, synthesizing clinical characteristics and radiological features acquired from CT scans, significantly augmented the accuracy of prognosticating chronic hydrocephalus in individuals with aSAH. This novel tool holds substantial promise in assisting healthcare professionals in formulating precise and individualized treatment strategies, offering accurate predictions concerning chronic hydrocephalus among aSAH patients.

## Data availability statement

The raw data supporting the conclusions of this article will be made available by the authors, without undue reservation.

## Ethics statement

The studies involving humans were approved by Affiliated People’s Hospital of Jiangsu University. The studies were conducted in accordance with the local legislation and institutional requirements. The ethics committee/institutional review board waived the requirement of written informed consent for participation from the participants or the participants’ legal guardians/next of kin because this is a Retrospective Cohort Study. Written informed consent was obtained from the individual(s) for the publication of any potentially identifiable images or data included in this article.

## Author contributions

DR: Data curation, Writing – original draft. LY: Software, Writing – original draft. XN: Data curation, Writing – original draft. LS: Data curation, Software, Writing – original draft. QY: Writing – original draft. LZ: Writing – original draft. ZZ: Writing – original draft. CY: Writing – original draft. CB: Writing – original draft. SX: Writing – review & editing. SE: Funding acquisition, Software, Writing – original draft, Writing – review & editing.
